# Procrastination and online social anxiety: the mediating role of cognitive overload and the moderating influence of mindfulness

**DOI:** 10.3389/fpubh.2026.1710399

**Published:** 2026-01-27

**Authors:** Shaolei Chen, Zhuoling Xie, Hongjing Wu, Zichuan Song

**Affiliations:** 1 Software Engineering Institute of Guangzhou, Guangzhou, China; 2 City University of Macau, Taipa, Macao SAR, China; 3 Fudan University, Shanghai, China

**Keywords:** cognitive overload, Conservation of Resources theory, mindfulness, online social anxiety, procrastination

## Abstract

In an era of ubiquitous digital communication, online social anxiety has become an increasingly salient issue in the context of digital wellbeing. While procrastination is a known risk factor for psychological distress, the cognitive pathways linking it to online anxiety—and the protective factors that might mitigate these associations—remain poorly understood. Drawing on Conservation of Resources (COR) theory, this study examines a theorized “depletion–overload–anxiety” pathway by testing whether cognitive overload mediates the association between procrastination and online social anxiety, and whether mindfulness buffers this process. A cross-sectional survey of 580 adults measured procrastination in online social contexts, cognitive overload, online social anxiety, and trait mindfulness. The results showed a significant positive association between procrastination and online social anxiety, which was partially mediated by cognitive overload. Mindfulness functioned as a protective resource, moderating both the link between procrastination and cognitive overload and the direct association with anxiety; specifically, higher mindfulness attenuated the adverse associations of procrastination with both overload and anxiety. These findings suggest that procrastination may be related to elevated online social anxiety partly through increased cognitive overload, whereas mindfulness may buffer this association by supporting more adaptive attentional regulation. By framing procrastination-related strain in digital communication as a digital wellbeing issue, the study speaks to public digital health efforts aimed at reducing technology-related stress and improving psychological functioning in everyday online interaction. Given the cross-sectional design, the findings should be interpreted as patterns of association consistent with the proposed model rather than causal effects. Accordingly, digital wellbeing initiatives may consider incorporating mindfulness-based strategies to help reduce the psychological strain associated with procrastination in digital communication settings.

## Introduction

**Figure 1:**
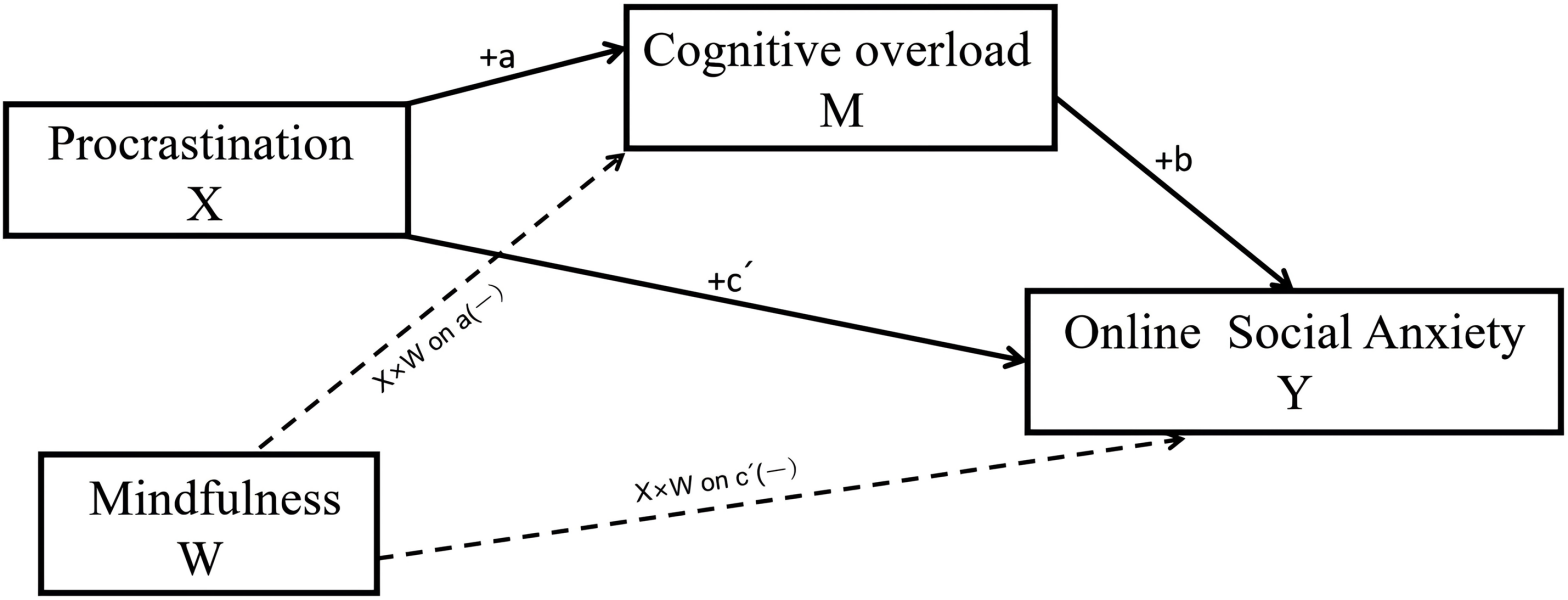
Proposed moderated mediation model (PROCESS Model 8).

Online social interaction has become integral to contemporary life, yet it also introduces distinct psychological burdens. Online social anxiety (OSA)-a heightened sense of tension and unease experienced in virtual social contexts (1)-is particularly sensitive to digital stressors such as “always-on” availability (2). Recent evidence suggests that OSA not only undermines the quality of social exchange but is linked to broader psychological distress, highlighting its public health relevance. From a public digital health perspective, anxiety in online social interaction is not only an individual psychological experience but also a population-level concern because digital communication has become a default channel for work coordination, social support, and civic participation. When online interaction repeatedly triggers tension, worry, or avoidance, individuals may withdraw from beneficial social ties, experience sustained stress, and show poorer daily functioning. Understanding modifiable cognitive pathways that connect everyday digital behaviors (e.g., delayed replying and notification avoidance) to online social anxiety can therefore inform scalable digital wellbeing approaches, including brief self-regulation tools and platform-level design strategies. In parallel, mHealth applications have increasingly been used to deliver scalable support for mental wellbeing, including interventions targeting affective distress in everyday life (3), suggesting a feasible delivery pathway for brief, low-cost digital wellbeing components. Procrastination, a common manifestation of self-regulatory failure, is especially salient in online social contexts: individuals delay replying to messages, postpone group participation, and defer handling notifications (4). Such delays are often amplified by the affordances of digital media-such as asynchronous communication, notification bursts, and socially visible responsiveness norms-which can turn minor postponements into an accumulating backlog of unprocessed social information and pending interaction tasks. These behaviors elevate cognitive demands and social stress (5). Prior work on social networking sites similarly suggests that information and social demands can accumulate into a sense of overload, which is associated with fatigue and reduced wellbeing (6, 7). Related research also suggests that excessive or problematic social media use can consume time and attention and undermine individuals’ functioning by depleting psychological resources (8). From the perspective of Conservation of Resources (COR) theory, procrastination depletes core resources-time and attention-creating a “resource debt” that facilitates subsequent stress responses (9). This study is conducted in the Chinese cultural context, where online social behavior is embedded in relational norms. Face concerns and collectivistic expectations may heighten sensitivity to responsiveness and perceived evaluation in online interactions, potentially shaping the intensity of OSA and the perceived consequences of delayed responding. As a result, delayed responding may be experienced as more socially costly, which could strengthen both the procrastination–OSA link and the anxiety implications of overload. Clarifying this cultural backdrop helps readers interpret the boundary conditions of the findings and their potential generalizability. Against this backdrop, we propose a moderated mediation model to examine how procrastination contributes to OSA via cognitive overload, and whether mindfulness buffers this pathway. Cognitive overload-when information-processing demands exceed capacity (10)—is common in high-frequency social media environments. We posit that procrastination increases cognitive demands by allowing unresolved social information to accumulate, thereby increasing the risk of cognitive overload and, in turn, intensifying OSA. Conversely, mindfulness-as nonjudgmental present-moment awareness (11)-may mitigate this chain by optimizing attentional allocation and reducing rumination (12, 13). Conceptually, the overload component is also consistent with cognitive load theory, which emphasizes capacity limits under high demands and frequent task switching (14). In addition, procrastination may be experienced as reduced autonomy and competence when individuals feel controlled by external interaction demands, which can further contribute to anxiety in evaluative contexts (15). In addition to addressing an increasingly important digital wellbeing concern, this study contributes in three ways. First, we conceptualize procrastination in online social interaction as a context-specific self-regulatory failure that can accumulate unresolved social tasks (e.g., pending replies and notifications), thereby increasing perceived cognitive demands. Second, drawing on COR theory, we specify cognitive overload as a plausible mechanism through which procrastination may be linked to online social anxiety. Third, we examine mindfulness as a protective resource that may buffer both the buildup of cognitive overload and the direct procrastination–anxiety association, offering practical implications for interventions aimed at promoting healthier online communication habits. Accordingly, using PROCESS Model 8 and controlling for demographic covariates, we test (1) the positive association between procrastination and OSA; (2) the mediating role of cognitive overload; and (3) the negative moderating effects of mindfulness on the procrastination → overload and procrastination → OSA paths, as well as the attenuation of the indirect effect at higher levels of mindfulness (Figure 1).

## Theoretical framework and literature review

### Theoretical foundation

We anchor our model in Conservation of Resources (COR) theory, which posits that individuals strive to acquire, retain, and protect valued resources and experience stress when these resources are threatened, lost, or insufficient to meet situational demands (9). In digitally mediated social contexts, three classes of resources are particularly salient: time, attention, and cognitive capacity. These resources are continuously taxed by message streams, notification-driven interruptions, and implicit norms of timely responsiveness. Accordingly, COR theory provides a useful lens for conceptualizing how everyday online behaviors can translate into resource strain and, ultimately, psychological distress. In the present model, procrastination in online social interaction is treated as a resource-depleting pattern. Specifically, delaying socially relevant tasks (e.g., postponing replies and deferring interaction-related decisions) may create a “resource debt” by allowing unfinished social obligations to accumulate. This backlog can keep interaction demands cognitively accessible, repeatedly drawing attention back to pending messages and increasing perceived time pressure. Over time, such persistent attentional capture and task accumulation may elevate cognitive overload, defined here as a perceived capacity–demand mismatch in information processing (10, 16). Cognitive overload is further consistent with cognitive load theory, which emphasizes the limits of working memory and the overload risk posed by interruptions and task switching—features that are common in notification-driven online interaction (14). When individuals experience overload, they may find it more difficult to filter information, prioritize responses, and maintain effective online communication, which can increase negative appraisal and perceived social risk. Within this resource framework, online social anxiety (OSA) is conceptualized as an affective stress response that arises when individuals perceive inadequate resources to cope with online interaction demands that carry social risk. In addition to evaluation-related concerns, online interaction may also evoke anxiety through perceived threats to informational control (e.g., privacy leakage and unintended exposure), which is reflected in the privacy-concerns facet of the OSA measure used in this study. Under resource strain, people may become more vigilant to potential social mistakes, misinterpret ambiguous feedback, or worry about negative evaluation, thereby experiencing heightened anxiety in online social settings. In parallel, we also draw on a self-determination perspective to acknowledge that persistent postponement under externally imposed interaction demands may frustrate autonomy and competence needs, which can further intensify anxiety in evaluative contexts (15). By contrast, mindfulness is conceptualized as a trait-like protective resource that supports attentional regulation and more adaptive responding to internal experiences. Through present-moment awareness and reduced ruminative reactivity, mindfulness may help individuals allocate attention more deliberately and disengage from self-critical rumination, thereby reducing the likelihood that procrastination escalates into cognitive overload and anxiety (17, 18). Building on COR theory, we therefore propose a depletion–overload–anxiety pathway in which procrastination is associated with higher OSA partly via increased cognitive overload, and mindfulness buffers this process by attenuating (a) the procrastination → overload association and (b) the direct procrastination → OSA association. Accordingly, we next develop hypotheses regarding (1) the overall association between procrastination and OSA, (2) the mediating role of cognitive overload, and (3) the moderating role of mindfulness on both the a-path and the direct path, as well as on the conditional indirect effect.

### The nexus between procrastination and online social anxiety

Procrastination refers to the voluntary postponement of intended actions even when one anticipates negative outcomes (19). In online contexts, it takes the form of delayed replies, deferred participation in group chats, or avoided notification checking, which can diminish interaction quality and reciprocity. From a resource-based view, postponement keeps unfinished social tasks cognitively active, repeatedly drawing attention to pending messages and generating anticipatory tension about future responding (20, 21). Such dynamics heighten perceived threat and avoidance, increasing OSA.

*Hypothesis 1 (H1):* Procrastination is positively associated with online social anxiety.

### The mediating role of cognitive overload

Cognitive overload refers to a state in which information-processing demands exceed an individual’s capacity (16). Social media environments-with high message velocity, multitopic threads, and frequent reciprocal demands-are fertile ground for overload (10). Procrastination aggravates this exposure by allowing unresolved messages and social obligations to accumulate, forcing multi-threaded processing under time pressure and reducing decision efficiency. Overload, in turn, impairs information filtering and perspective-taking, amplifies negative interpretation, and reduces perceived social efficacy-mechanisms strongly tied to anxiety in evaluative social contexts.

*Hypothesis 2 (H2):* Cognitive overload mediates the relationship between procrastination and online social anxiety.

### The moderating role of mindfulness

Mindfulness-nonjudgmental, present-moment awareness-improves attentional control and emotion regulation (17, 22). In the current framework, although mindfulness may be associated with lower overload and anxiety, our model focuses on its buffering role in weakening the adverse pathways from procrastination to overload and anxiety. Specifically, mindfulness is expected to regulate resource dynamics along the procrastination paths. First, mindfulness should weaken the a-path (procrastination → overload) by enabling timely triage (e.g., prioritizing essential messages), reducing self-criticism about delays, and limiting rumination-thus preventing backlog escalation (23). Second, mindfulness should buffer the direct c′-path (procrastination → OSA) by reframing anticipated social consequences and reducing catastrophic appraisals, thereby conserving cognitive resources for interaction. As a result, the indirect effect of procrastination on OSA via overload should be weaker at higher levels of mindfulness.

*Hypothesis 3a (H3a):* Mindfulness negatively moderates the relationship between procrastination and cognitive overload (a-path).

*Hypothesis 3b (H3b):* Mindfulness negatively moderates the direct relationship between procrastination and online social anxiety (c′-path).

*Hypothesis 3c (H3c):* The indirect effect of procrastination on online social anxiety via cognitive overload is weaker at higher levels of mindfulness (index of moderated mediation < 0).

Procrastination (X) positively predicts cognitive overload (M) and online social anxiety (Y); cognitive overload positively predicts online social anxiety. Mindfulness (W) negatively moderates the a-path (X → M) and the direct effect c′ (X → Y). Dashed lines indicate moderation (interaction effects) on the corresponding paths rather than direct main effects.

### Research method

This study employed a convenience sampling approach and collected data via an online survey platform. A total of 580 valid questionnaires were obtained. Data were collected using Wenjuanxing and distributed through WeChat to recruit active social media users across China. The sample was diverse in terms of gender, age, education, and employment status (Male: 52.07%; Female: 47.93%; Age: 18–25 = 35.17%, 26–35 = 42.24%, 36–45 = 16.38%, ≥46 = 6.21%; Education: high school or below = 8.62%, associate degree = 15.52%, bachelor’s degree = 56.03%, master’s degree or above = 19.83%). Incomplete responses were excluded. All participants provided online informed consent. To enhance data quality in the online survey setting, we excluded incomplete responses and implemented basic screening procedures embedded in the questionnaire (e.g., attention-check items). Participation was anonymous, and respondents were informed that there were no right or wrong answers to reduce evaluation concerns and encourage honest reporting.

#### Scoring and item transparency

Unless otherwise noted, all constructs were operationalized as mean scores (range = 1–5), with higher scores indicating higher levels of the corresponding construct. To facilitate transparency and replication, Table 1 provides the full wording of all measurement items used in the questionnaire, along with item codes and sources. Reverse-scored mindfulness items were recoded prior to computing composite scores.

**Table 1:** Measurement items, response formats, and sources.

Variable	Items	Resources
Procrastination *online social contexts*	P1 When faced with online social messages that require an immediate response, I often procrastinate.	Adapted from Lay (24)
	P2 I frequently delay completing tasks related to managing information on online social platforms.	
	P3 I procrastinate registering for or preparing for online social events.	
	P4 I consistently delay responding to requests for online social interaction.	
	P5 I tend to postpone complex communication tasks in online social networks.	
	P6 I often put off matters related to online social activities until the last minute.	
	P7 I deliberately procrastinate checking notifications on online social platforms.	
	P8 I consistently delay responding to online social messages.	
Online social anxiety (OSA) *Evaluation fear*	OSA-E1 After posting content on social media, I repeatedly worry about others’ evaluations.	Adapted from Wang et al. (25)
	OSA-E2 I am afraid of being negatively evaluated by others after speaking in group chats.	
	OSA-E3 I worry that others will think my online comments are stupid.	
	OSA-E4 I feel anxious that my posts will not receive enough likes or positive feedback.	
	OSA-E5 I worry about being laughed at or mocked in online discussions.	
	OSA-E6 I fear that my opinions will be criticized or attacked by others online.	
	OSA-E7 I worry that my online profile or activities will be judged unfavorably by others.	
	OSA-E8 I worry about making social mistakes (e.g., typos, wrong tags) that others will notice.	
Online social anxiety (OSA) *Privacy concerns*	OSA-P1 I worry about the leakage of personal information in online social interactions.	Same as above
	OSA-P2 I worry about overexposure of privacy in online social interactions.	
	OSA-P3 I feel uneasy about how much personal data social media platforms collect.	
	OSA-P4 I worry that my private messages could be seen by unintended people.	
	OSA-P5 I worry that my location or activity status might be tracked through social apps.	
	OSA-P6 I feel anxious about the permanent digital footprint I am creating.	
Online social anxiety (OSA) *Interaction anxiety*	OSA-I1 I often feel nervous and uneasy when participating in online video conferences.	Same as above
	OSA-I2 I feel anxious when socializing online with unfamiliar people.	
	OSA-I3 In real-time online chats, I feel pressured to respond immediately.	
	OSA-I4 I get nervous when I have to speak up in a large online group.	
	OSA-I5 I feel uncomfortable initiating a conversation with someone online.	
	OSA-I6 I worry about running out of things to say during an online conversation.	
Cognitive overload	CO1 I find it difficult to process too much information on social media.	Adapted from Karr-Wisniewski and Lu (10)
	CO2 The sheer volume of information makes it difficult for me to filter out key content.	
	CO3 Due to the large volume of information, I often struggle to process online social information effectively.	
	CO4 Information updates too quickly, overwhelming me.	
	CO5 Maintaining online social relationships (e.g., replying to messages, liking interactions) puts me under a lot of pressure.	
	CO6 The multi-threaded interactions in online social media drain my energy.	
	CO7 Handling multiple online social tasks simultaneously is difficult for me to cope with.	
	CO8 Frequent interactions in online social media cause me cognitive fatigue.	
Mindfulness	MF1 I pay attention to what I’m doing.	Selected from the Five Facet Mindfulness Questionnaire [FFMQ Baer et al. (22)]
	MF2 I notice my feelings without getting carried away by them.	
	MF3 I am aware of my thoughts and feelings as they happen.	
	MF4 (R) I find myself doing things without paying attention.	
	MF5 When I have upsetting thoughts or images, I can let them go without getting caught up in them.	
	MF6 I notice physical sensations, such as the wind in my hair or sun on my face.	
	MF7 I am good at finding words to describe my feelings.	
	MF8 (R) I criticize myself for having irrational or inappropriate emotions.	
	MF9 I perceive my feelings and emotions without having to react to them.	
	MF10 (R) I tell myself I should not be feeling the way I’m feeling.	
	MF11 I notice how foods and drinks affect my thoughts, bodily sensations, and emotions.	
	MF12 (R) I rush through activities without being really attentive to them.	
	MF13 (R) When I do things, my mind wanders off and I’m easily distracted.	
	MF14 (R) I think some of my emotions are bad or inappropriate and I should not feel them.	
	MF15 I watch my feelings without getting lost in them.	

This table reports the full wording of all items used in the questionnaire and their sources. Procrastination and cognitive overload items were contextualized to online social scenarios by two PhD candidates and reviewed by three experts in social psychology. Measures originally developed in English were translated using a translation–back-translation procedure and pilot-tested for clarity. Items were rated on 5-point scales, with response anchors reported in the Research Method section. Items marked (*R*) in the mindfulness scale were reverse-coded prior to analysis.

#### Procrastination (online social contexts)

Procrastination was measured with eight items adapted from (24) and contextualized to online social interaction tasks (Table 1, P1–P8). Responses were recorded on a 5-point scale (1 = completely inconsistent, 5 = completely consistent), and item scores were averaged to create a composite procrastination score (range = 1–5). For example, P1 captures procrastination under immediate reply demands, whereas P7 reflects intentional delay in checking platform notifications; together they represent postponement in everyday digital communication.

#### Online social anxiety (OSA)

OSA was assessed using a 20-item scale adapted and localized from Wang et al. (25), comprising three dimensions: evaluation fear (8 items), privacy concerns (6 items), and interaction anxiety (6 items) (Table 1, OSA-E1–OSA-I6). Responses were recorded on a 5-point Likert scale (1 = strongly disagree, 5 = strongly agree), and items were averaged to compute an overall OSA score (range = 1–5). For illustration, OSA-E1 reflects worry about social evaluation after posting, whereas OSA-P1 captures anxiety about personal-information leakage during online interaction. A confirmatory factor analysis (CFA) supported acceptable model fit (*χ*^2^/df = 4.05, CFI = 0.92, TLI = 0.91, SRMR = 0.07, RMSEA = 0.07).

#### Cognitive overload

Cognitive overload was measured using eight items adapted from Karr-Wisniewski and Lu (10) and contextualized to social media, covering information load (4 items) and social load (4 items) (Table 1, CO1–CO8). Responses were recorded on a 5-point Likert scale (1 = strongly disagree, 5 = strongly agree). For example, CO1 represents difficulty processing excessive information volume, whereas CO5 reflects perceived pressure from maintaining online relationships (e.g., replying and liking). Items were averaged to compute an overall cognitive overload score (range = 1–5). CFA indicated acceptable fit (*χ*^2^/df = 4.78, CFI = 0.95, TLI = 0.93, SRMR = 0.04, RMSEA = 0.08).

#### Mindfulness (trait)

Trait mindfulness was assessed using a 15-item set selected from the Five Facet Mindfulness Questionnaire (FFMQ; Baer et al. (22)) (Table 1, MF1–MF15). Items were translated using a translation–back-translation procedure and were not substantively reworded to preserve content validity. Responses were recorded on a 5-point frequency scale (1 = never, 5 = always). For example, MF1 reflects attentional awareness of ongoing activity, whereas MF5 captures non-reactivity to distressing thoughts or images. Reverse-coded items were recoded prior to analysis, and items were averaged to compute a composite mindfulness score (range = 1–5). CFA indicated acceptable model fit (*χ*^2^/df = 3.05, CFI = 0.93, TLI = 0.91, SRMR = 0.05, RMSEA = 0.06).

### Statistical analysis

All analyses were conducted in SPSS 25.0 using the PROCESS Model 8 (26). Model 8 was selected because it allows us to test the mediation pathway (procrastination → cognitive overload → online social anxiety) while simultaneously examining whether mindfulness moderates the procrastination → cognitive overload path and the direct procrastination → online social anxiety path. Continuous predictors (procrastination and mindfulness) were mean-centered prior to constructing interaction terms to facilitate interpretation and reduce nonessential multicollinearity. We report unstandardized coefficients (b), standard errors (SE), and 95% bias-corrected bootstrap confidence intervals based on 5,000 resamples. For indirect and conditional indirect effects, statistical support was inferred when the bootstrap confidence interval did not include zero. Because all variables were computed as mean scores (range = 1–5), coefficients can be interpreted in the original mean-score metric.

### Common method bias (CMB)

Because all variables were assessed via self-report questionnaires at a single time point, common method variance cannot be fully ruled out. To mitigate this concern, we implemented procedural remedies (e.g., use of reverse-coded items and attention checks, as well as clear instructions and anonymized responding) and conducted statistical checks. Specifically, we performed Harman’s single-factor test, in which the first factor accounted for 28.43% of the total variance. As an additional robustness check, we estimated a CFA model including a common latent factor; the substantive conclusions regarding the focal paths were not materially altered (27).

### Covariates and multicollinearity

Gender, age, education, and employment status were included as covariates in all models to account for potential demographic differences in online communication behaviors and psychological experiences. Gender was assessed with three response options (male, female, prefer not to say); no participant selected “prefer not to say,” and gender was therefore coded as a binary covariate. Age and education were entered as ordinal covariates based on the response categories in the questionnaire (coded in ascending order). Employment status was dummy-coded with “employed” as the reference category (student, freelancer/self-employed, and unemployed/other). Variance inflation factors (VIFs) ranged from 1.35 to 1.78, indicating no serious multicollinearity concerns.

## Results

### Descriptive statistics and correlations

As shown in Table 2, all variables were computed as mean scores (1–5). Procrastination was positively correlated with cognitive overload (*r* = 0.42, *p* < 0.001) and online social anxiety (*r* = 0.33, *p* < 0.001). Cognitive overload was also positively correlated with online social anxiety (*r* = 0.47, *p* < 0.001). Mindfulness was negatively correlated with procrastination (*r* = −0.40, *p* < 0.001), cognitive overload (*r* = −0.22, *p* < 0.001), and online social anxiety (*r* = −0.23, *p* < 0.001). Overall, the correlation patterns were consistent with the hypothesized directions (Table 2), supporting the subsequent moderated mediation analyses.

**Table 2:** Descriptive statistics and correlation matrix of core variables (*N* = 580).

Variable	*M*	SD	1	2	3	4
1. Procrastination	3.05	1.05	—	0.42***	0.33***	−0.40***
2. Cognitive overload	3.10	0.80	0.42***	—	0.47***	−0.22***
3. Online social anxiety	3.06	0.70	0.33***	0.47***	—	−0.23***
4. Mindfulness	3.20	1.15	−0.40***	−0.22***	−0.23***	—

*M* = mean; SD = standard deviation. Entries are Pearson’s correlation coefficients (two-tailed). ***p < 0.001. All constructs were operationalized as mean scores (range = 1–5). Higher scores indicate higher levels of procrastination, cognitive overload, and online social anxiety, whereas higher mindfulness scores indicate greater trait mindfulness.

### Assessment of convergent and discriminant validity

Convergent validity was evaluated using average variance extracted (AVE) for the constructs with CFA measurement models (i.e., online social anxiety, cognitive overload, and mindfulness). As shown in Table 3, AVE values were above 0.50 for these CFA-assessed constructs, indicating adequate convergent validity (28). The square roots of AVE (bold diagonals) were greater than the inter-construct correlations (off-diagonals), providing support for discriminant validity among these constructs. Procrastination was treated as a composite observed score in the subsequent PROCESS analyses and therefore was not included in the AVE-based validity assessment.

**Table 3:** AVE-based convergent and discriminant validity for CFA-assessed constructs.

Variable	AVE	1	2	3
1. Cognitive overload	0.549	**0.741**		
2. Online social anxiety	0.536	0.470	**0.732**	
3. Mindfulness	0.567	−0.220	−0.230	**0.753**

AVE = average variance extracted. Bold diagonal values are the square roots of AVE. Off-diagonal values are Pearson’s correlations (two-tailed) based on the mean scores of each construct. AVE values were derived from the corresponding CFA measurement models. Procrastination was not included in this AVE-based assessment because it was treated as a composite observed score in the main analyses.

### Moderated mediation analysis

Overall, the results were consistent with the proposed moderated mediation model (H1–H3). We first tested the mediation model (Table 4). Procrastination positively predicted online social anxiety (*b* = 0.27, 95% CI [0.19, 0.35]) and cognitive overload (*b* = 0.42, 95% CI [0.35, 0.49]), and cognitive overload positively predicted online social anxiety (*b* = 0.31, 95% CI [0.23, 0.39]). The indirect effect via cognitive overload was supported (*ab* = 0.13, 95% bootstrap CI [0.07, 0.20]), as the confidence interval did not include zero, supporting H1 and H2.

**Table 4:** Results of the mediation model.

Paths	*b*	SE	LLCI	ULCI	*p*
Direct effect					
Procrastination → Online social anxiety	0.27	0.04	0.19	0.35	***
Procrastination → Cognitive overload	0.42	0.03	0.35	0.49	***
Cognitive overload → Online social anxiety	0.31	0.04	0.23	0.39	***
Total effect					
Procrastination → Online social anxiety	0.40	0.04	0.32	0.48	***
Indirect effect					
Procrastination → Cognitive overload → Online social anxiety	0.13	0.03	0.07	0.20	–

N = 580. The table presents unstandardized coefficients (b) with standard errors (SE) and 95% bias-corrected bootstrap confidence intervals (CI) based on 5,000 resamples. LLCI and ULCI denote the lower and upper limits of the CI, respectively. All models control for gender, age, education, and employment status. Asterisks indicate statistical significance: *p < 0.05, **p < 0.01, ***p < 0.001. For indirect effects, significance is inferred when the 95% CI does not include zero; thus, p-values are omitted and denoted by “—” in the table. Coefficients are interpretable on the original 1–5 mean-score metric.

We then tested the moderated mediation model using PROCESS Model 8 (26), controlling for demographic covariates (Table 5). The procrastination × mindfulness interaction negatively predicted cognitive overload (*b* = −0.15, 95% CI [−0.20, −0.04]), supporting H3a, and it also negatively predicted online social anxiety in the outcome equation (*b* = −0.078, 95% CI [−0.17, −0.01]), supporting H3b. These negative interaction terms indicate a buffering pattern, such that the positive associations between procrastination and both cognitive overload and online social anxiety were weaker at higher levels of mindfulness. Consistent with this interpretation, conditional indirect effects via cognitive overload decreased as mindfulness increased (low: 0.18; mean: 0.13; high: 0.08), and the index of moderated mediation was negative with a confidence interval that excluded zero (Index = −0.046, 95% CI [−0.10, −0.02]), supporting H3c.

**Table 5:** Results of the moderated mediation analysis.

Paths	*b*	SE	LLCI	ULCI	*p*
Cognitive overload (M)					
Procrastination → Cognitive overload	0.42	0.06	0.30	0.54	***
Mindfulness → Cognitive overload	−0.26	0.05	−0.36	−0.16	***
Procrastination × mindfulness → Cognitive overload	−0.15	0.04	−0.20	−0.04	**
Online social anxiety (Y)					
Procrastination → Online social anxiety	0.27	0.04	0.19	0.35	***
Mindfulness → Online social anxiety	−0.15	0.04	−0.23	−0.07	***
Procrastination × mindfulness → Online social anxiety	−0.078	0.04	−0.17	−0.01	*
Cognitive overload → Online social anxiety	0.31	0.04	0.23	0.39	***
Conditional effects of procrastination on cognitive overload (a-path)					
Low mindfulness (*M* − SD)	0.59	0.08	0.43	0.75	***
Medium mindfulness (*M*)	0.42	0.06	0.30	0.54	***
High mindfulness (*M* + SD)	0.25	0.07	0.11	0.39	**
Conditional direct effects of procrastination on online social anxiety (c′-path)					
Low mindfulness (*M* − SD)	0.35	0.07	0.21	0.49	***
Medium mindfulness (*M*)	0.27	0.04	0.19	0.35	***
High Mindfulness (*M* + SD)	0.18	0.06	0.06	0.30	**
Conditional indirect effects (Procrastination → Cognitive overload → Online social anxiety)					
Low mindfulness (*M* − SD)	0.18	0.04	0.11	0.26	–
Medium mindfulness (*M*)	0.13	0.03	0.07	0.20	–
High mindfulness (*M* + SD)	0.08	0.03	0.02	0.14	–
Index of moderated mediation	−0.046	0.02	−0.10	−0.02	–

*N* = 580. Unstandardized coefficients (*b*) are reported with standard errors (SE) and 95% bias-corrected bootstrap confidence intervals (5,000 resamples). LLCI = lower limit; ULCI = upper limit of the 95% confidence interval. Continuous predictors (procrastination and mindfulness) were mean-centered prior to creating interaction terms. Therefore, the coefficient for procrastination in the cognitive overload equation represents the conditional effect when mindfulness is at its mean (*W* = 0), corresponding to the “medium mindfulness (*M*)” simple slope. Covariates (gender, age, education, and employment status) were included in all models. For conditional indirect effects and the index of moderated mediation, statistical support is inferred when the bootstrap confidence interval does not include zero; therefore, *p*-values are not reported for these effects (shown as “–”). *p < 0.05, **p < 0.01, ***p < 0.001 (for regression coefficients). Coefficients can be interpreted in the original mean-score metric (range = 1–5).

**Figure 2:**
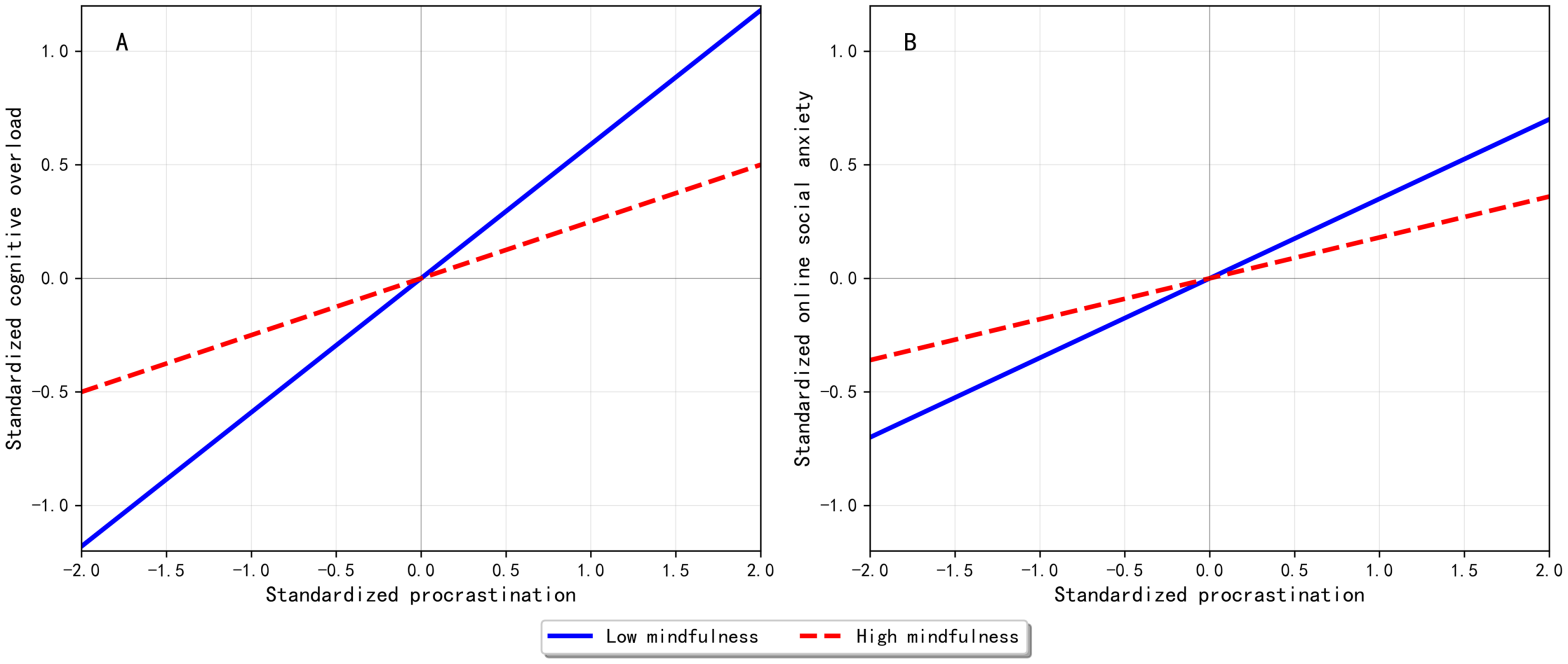
Simple slope plot for the moderating role of mindfulness. Panel **(A)** shows the conditional effect of procrastination on cognitive overload (X→M) at low (M − 1 SD) and high (M + 1 SD) levels of mindfulness. Panel **(B)** shows the conditional effect of procrastination on online social anxiety (X→Y) at low and high levels of mindfulness.

To interpret the significant interaction terms, we conducted simple slope analyses by estimating the conditional effects of procrastination at low (*M* − 1 SD) and high (*M* + 1 SD) levels of mindfulness and plotted the results in Figure 2. Across both panels, the slopes remained positive but were consistently steeper under low mindfulness, indicating that the associations between procrastination and both cognitive overload and online social anxiety were weaker at higher levels of mindfulness. We further examined conditional indirect effects via cognitive overload across mindfulness levels and the index of moderated mediation to assess whether the indirect association between procrastination and online social anxiety varied as a function of mindfulness.

Panel A shows the conditional effect of procrastination on cognitive overload (X → M) at low (M − 1 SD) and high (*M* + 1 SD) levels of mindfulness. Panel B shows the conditional effect of procrastination on online social anxiety (X → Y) at low and high levels of mindfulness.

Further examination of conditional indirect effects (Table 5) showed that the indirect effect of procrastination on online social anxiety via cognitive overload decreased as mindfulness increased (low mindfulness: 0.18; mean mindfulness: 0.13; high mindfulness: 0.08). These conditional indirect effects were supported, as their 95% bias-corrected bootstrap confidence intervals did not include zero, which is consistent with H3c.

## Discussion

Building on these results, we discuss a potential psychological pathway linking procrastination in online social contexts to online social anxiety, as well as the buffering role of mindfulness. Overall, the pattern of findings was consistent with a “depletion–overload–anxiety” pathway and suggested that mindfulness may attenuate both the buildup of cognitive overload and the association between procrastination and online social anxiety. This interpretation is also compatible with evidence that overload-like experiences in social media use (e.g., fatigue from continuous information and social demands) are associated with poorer psychological outcomes, including increased distress indicators (7, 29).

The findings are consistent with prior research suggesting that self-regulatory difficulties in digital environments can carry psychological costs, and they extend this work by highlighting cognitive overload as a plausible mechanism linking procrastination to online social anxiety. In the Chinese cultural context, responsiveness norms and face-related concerns may increase the perceived social consequences of delayed replying, making pending online interactions more salient and potentially more anxiety-provoking. This interpretation does not imply cultural determinism; rather, it highlights a contextual boundary condition that warrants cross-cultural replication. Procrastination was positively associated with cognitive overload, which in turn was associated with higher online social anxiety. From the perspective of Conservation of Resources (COR) theory, procrastination may contribute to a form of “cognitive debt,” reflected in a perceived backlog of unresolved online social tasks and information that continues to occupy attention and working memory. As this backlog accumulates, individuals may find it more difficult to prioritize, filter, and respond effectively during online interactions. In socially evaluative digital environments, heightened overload may coincide with increased worry about making mistakes, being judged, or responding inappropriately, which may correspond to higher levels of online social anxiety.

From a public digital health standpoint, the pattern observed here suggests that everyday digital communication behaviors (e.g., postponing replies and leaving interaction tasks unresolved) may coincide with measurable psychological strain, partly through heightened cognitive overload. This points to at least two intervention entry points that are scalable: (a) reducing the accumulation of pending interaction tasks by promoting brief, practical self-regulation routines; and (b) strengthening attentional regulation so that users can engage with online demands without becoming cognitively overwhelmed. Indeed, app-delivered contemplative practices delivered via short-form video have been shown to improve mindfulness-related outcomes and other psychological indicators in young adult populations (30), supporting the feasibility of lightweight, scalable delivery formats.

### Theoretical and practical implications

#### Theoretical implications

This study contributes to research on digital wellbeing by extending COR theory to digitally mediated social interaction and by framing procrastination in online social contexts as a resource-depleting pattern that is linked to cognitive overload and, subsequently, online social anxiety. By integrating the notion of overload as a capacity–demand mismatch, the findings help clarify why procrastination may be psychologically taxing in high-frequency communication environments where attention is repeatedly interrupted and interaction demands accumulate.

#### Practical implications

The findings suggest that digital wellbeing initiatives may benefit from going beyond time-management advice and incorporating mindfulness-based strategies that support attentional regulation in everyday online communication. In addition to individual-level approaches, platform-level design features (e.g., summary notifications, batching updates, or structured message prioritization) may help reduce information burden and support users’ psychological wellbeing. From a human factors perspective, interface-level presentation (e.g., iconography and visual motion cues) can shape how efficiently users interpret and act on information, thereby influencing cognitive demand during interaction (31).

To translate these implications into applied digital wellbeing contexts, we outline several mindfulness-informed components aligned with the observed associations:

(1) Micro-mindfulness practices for resource recovery. Brief practices (1–3 min), such as focused breathing or body scanning before opening social media apps, may help users shift from automatic engagement to more deliberate interaction, potentially reducing initial cognitive load (32). Related evidence from occupational contexts suggests that brief meditation-based practices (e.g., loving-kindness meditation) can be integrated into everyday routines and may be associated with improved mindfulness-related and wellbeing outcomes (33).
(2) The “S.T.O.P.” technique for impulse control. To interrupt the cycle of procrastination and subsequent anxiety, the S.T.O.P. technique (Stop, Take a breath, Observe, Proceed) can be adapted to digital contexts. When users feel the urge to delay a reply or compulsively check notifications, applying this technique may help them observe discomfort without reacting automatically, thereby supporting non-judgmental awareness and more flexible self-regulation (17). Such brief, structured practices are also compatible with app-based delivery approaches reported in prior ISDT-oriented case studies, which can inform the design of micro-interventions embedded in routine digital use (33).
(3) Digital boundary setting as resource protection. This component integrates mindfulness principles with concrete behavioral strategies, such as establishing a “digital sunset” (a fixed time after which online social interactions cease) or systematically turning off non-essential notifications. These boundaries align with COR theory by proactively protecting attentional resources from constant digital interruptions, which may help reduce chronic cognitive overload over time (10). To enhance scalability and reach, such strategies could be delivered via mobile health (mHealth) applications that provide prompts, personalized boundary plans, and self-monitoring tools for individuals experiencing elevated online social anxiety. Given emerging evidence that mobile-delivered, short-form guided practices can produce measurable psychological benefits (30), these components may be adapted into scalable mHealth modules targeting online communication habits.

### Limitations

This study used a cross-sectional design; therefore, the proposed model should be interpreted as describing a correlational pattern that is consistent with the theorized pathway rather than providing causal evidence. In addition, the study relied on self-report measures collected at a single time point; future research could incorporate behavioral indicators (e.g., response latency logs) or multi-source assessments to strengthen inference. Future longitudinal, experimental, or intervention studies are needed to examine whether changes in procrastination or mindfulness are prospectively associated with changes in cognitive overload and online social anxiety. In addition, because the sample was drawn from China, cultural norms related to face concerns and responsiveness expectations may shape the strength of the observed associations. Cross-cultural replication would be valuable for assessing the generalizability of the findings and for identifying potential boundary conditions. In addition, the convenience sampling strategy may limit representativeness; future studies using probability-based or more diverse recruitment strategies would strengthen population-level inference. Incorporating objective behavioral indicators (e.g., response latency or notification-checking patterns) could also help clarify whether digitally observable behaviors correspond to the proposed cognitive pathway.

## Data Availability

The original contributions presented in the study are included in the article/supplementary material, further inquiries can be directed to the corresponding author.
